# An evaluation of Milligan-Morgan and Ferguson procedures for haemorrhoidectomy at Liaquat University Hospital Jamshoro, Hyderabad, Pakistan

**DOI:** 10.12669/pjms.291.2858

**Published:** 2013

**Authors:** Abdul Razaque Shaikh, Abdul Ghafoor Dalwani, Nasarullah Soomro

**Affiliations:** 1 Dr. Abdul Razaque Shaikh, Department of General Surgery, Liaquat University of Medical Health & Sciences Jamshoro, Sindh, Pakistan.; 2 Dr. Abdul Ghafoor Dalwani, MS, Department of General Surgery, Liaquat University of Medical Health & Sciences Jamshoro, Sindh, Pakistan.; 3 Dr. Nasuarullah Soomro, MBBS, Department of General Surgery, Liaquat University of Medical Health & Sciences Jamshoro, Sindh, Pakistan.

**Keywords:** Open haemorrhoidectomy, Closed haemorrhoidectomy, Complications

## Abstract

***Objective: ***o compare the outcome of Milligan-Morgan (MMH) and Ferguson (FH) techniques for haemorrhoidectomy with regard to postoperative pain, control of bleeding, early mobilization of patients and wound healing.

***Methodology:*** In this prospective, randomized clinical study conducted between January 2005 to December 2008, 213 patients with late 2^nd^ degree; third or fourth degree hemorrhoids were assigned to two groups. One hundred ten patients in group A were operated by an open method and 103 patients in group B were operated by closed method.

***Results:*** Age ranged from 22-70 years with mean age of 45.5 years. Peak incidence was between 41-50 years. Out of 213 patients, 170 (79.81%) were male and 43 (20.18%) were females. The mean ± SD operating time was significantly more in group B (31.3±4.8 min) than group A (25.2±5.6). The duration of hospitalization and duration off from work was more in group A than the group B. Wound healing was quicker in group B than the group A. Post operative pain scores were significantly low in the Group A than Group B during first 24 hours and at first bowel movements. Reactionary hemorrhage occurred in 4 (3.63%) patients of group A, no patient in group B developed this complication. Retention of urine was seen in 13 (11.81%) patients in group A and 4 (3.88%) in group B. No patient in group A developed anal stenosis, while 3 (2.91%) patients in group B developed anal stenosis. Wound infection was one (0.9%) in group A and two (1.9%) in group B. Two (3.63%) patients in group A came with recurrent hemorrhoids and in group B, only one (0.97%) patient reported recurrence.

***Conclusions:*** The closed technique is more beneficial with respect to postoperative pain, control of bleeding, early mobilization of patients and wound healing.

## Introduction

 Haemorrhoidectomy is believed to be an effectual, though agonizing cure of late 2^nd^ degree which did not respond to non surgical methods or third-degree and fourth degree hemorrhoids.^1^ It can be performed by the open or closed method.^2^^,^^3^ In Europe, the Milligan-Morgan method is more commonly in use, while in the United States the closed haemorrhoidectomy method, as illustrated by Ferguson and Heaton, is a common and traditional method.^2^^-^^4^ Closed haemorrhoidectomy is supposed to be less painful method and results in rapid wound healing.^2^^,^^5^ However, a disagreement is still there concerning these two techniques of management with regards to postoperative pain and complications.^4^^,^^6^^-^^8^

This study was designed to evaluate and compare the outcome of surgical repair of hemorrhoids by the open vs closed technique to assess the rate of postoperative complications in both procedures.

## Methodology

 This prospective, randomized clinical study was conducted in Surgical Unit III at Liaquat University Hospital Jamshoro Pakistan from January 2005 to December 2008, 213 patients with late 2^nd^ degree; third or fourth degree haemorrhoids were randomly assigned to two groups. Randomization was performed in Outpatient department. Patients in group A were operated by an open method and patients in group B were operated by closed method. Open haemorrhoidectomy was performed according to the Milligan-Morgan (MMH) and closed technique according to the Ferguson technique (FH).

 Patients with complicated, secondary, external haemorrhoids or associated with anorectal disorders were excluded from the study. Patients were evaluated by taking history, thorough general and local examination; digital rectal and proctoscopic examination. Patients were operated under spinal or general anesthesia. Bowel preparation was done by administering enema initially at night and subsequently next morning prior to surgery. All information was recorded on pre-designed proforma. Patients were explained about the two procedures and then informed consent was taken about inclusion in the trial.

 After induction of anesthesia, the procedures were performed keeping the patient in lithotomy position. Anus was dilated, but loco regional anesthetic blocks were not used. In 110 patients, Milligan Morgan’s technique (open) was used. The skin incision was made on the mucocutaneous border and haemorrhoids were excised to the anorectal junction with diathermy. The base of pedicle was transfixed with 2/0 polyglactin. The resulting wounds were left open and anal canal was plugged. In the other 103 cases Fergusons (closed) procedure was performed, vascular pedicle was high ligated with 2/0 polyglactin. After achieving the haemostasis the wound in the mucosa and skin was closed with 3/0 polyglactin.

 The pain score was evaluated by an independent blinded observer. A linear analogue scale was used, where 0 stand for no pain and 10 being worse pain ever experienced. The pain score was taken on 1^st^ postoperative day and than on first bowel movement. Out patient follow-up continued weekly until the wounds had completely healed. Wound healing was examined by insertion of a small anoscope well lubricated with lignocaine gel. Wound dehiscence was defined as any gaping of the wound whether in the anal canal or perianal skin.

***Statistical Analysis: ***Data were entered and analyzed in statistical program SPSS version 16.0. Qualitative data (frequencies and percentages) such as gender, degree of hemorrhoids, retention of urine, bleeding, wound healing, wound dehiscence, recurrence, anal stenosis, anal incontinences and wound infection etc. were presented as n(%) and chi square test was applied to compare the proportion between groups A(Open hemorrhoidectomy) and B (Closed hemorrhoidectomy). Numerical variables like age(in years), duration of symptoms (in years), operating time (in minutes), duration of hospitalization (in days), duration of off work (in days), healing time (in days), post operative pain score (duration first 24 hours, at first bowel movements) were presented as Mean + Standard Deviation (range) and student “t” test was used to compare the means (2 tailed) between two groups A(Open hemorrhoidectomy) and B (Closed hemorrhoidectomy). All the data were calculated on 95% confidence interval. A p value < 0.05 was considered as statistically significant level for all comparisons.

## Results

 A total of 213 patients were included in this study. Age ranged from 22-68 years with mean age of 45 years in Group A, and 46 years (24-70) in Group B. Out of 213 patients, 170 (79.81%) were male and 43 (20.18%) were females. In this study 3^rd^ degree haemorrhoids were more in male patients as compared to female patients. Treatment modalities used in this study were open and closed haemorrhoidectomy. In an open haemorrhoidectomy group A, out of 110 patients, 90 (81.81%) patients were males and 20 (18.18%) patients were females. In closed haemorrhoidectomy group B, out of 103 patients 80 (77.66%) patients were males and 23 (22.33%) patients were females (Table-I). It may be due to the fact that male patients seek advice early and female patients are reluctant to be examined by male surgeons. 

**Table-I:** Preoperative characteristics (n = 213).

	*Group A* *(Open hemorrhoidectomy)* *(Open hemorrhoidectomy)* *n = 110*	*Group B* *(Closed hemorrhoidectomy)* *n=103*	*P-value*
Age (in years)*	45.13 *+* 5.6 (22-68)	46.20 + 4.5 (24-70)	NS
Gender**			
Male female	90(81.8%) 20(18.1%)	80(77.6%) 23(22.3%)	NS
Degree of hemorrhoids**			
II III-IV	27(24.54%) 83(75.45%)	24(23.3%) 79(76.6%)	NS
Duration of symptoms (in years)*	9.0 + 0.1 (0.08-21)	10.1 + 0.3 (0.6-22)	NS

NS = Not significant

*Results are expressed as Mean + Standard Deviation (Range)

** Results are presented as n(%)

 In this study, patients presented with a variety of symptoms including bleeding per rectum, prolapse of mass per rectum, constipation, discharge, itching and anemia. The main complaint was bleeding and prolapse of mass per rectum i.e. 100% of cases. Bleeding was mostly in the form of streaming of drops in both groups. Constipation was also present in 78 patients (70.90%) in Group A, and 90 patients (87.37%) in Group B, P = 0.03. Other symptoms included discharge which was present in 71 patients (64.54%) in Group A, and 79 patients (76.69%) in Group B, P = 0.04. Itching was present in 76 patients (69.09%) in Group A, and 68 patients (66.01%) in Group B, P = 0.03. 

 Operating time was significantly more in group B (31.3+-4.8 min) than group A (25.2+-5.6). The duration of hospitalization and duration off from work was more in group A than the group B. Wound healing was quicker in group B than the group A (Table-II).

**Table-II:** Operating time, duration of hospitalization, duration off work, and healing time (n = 213).

	*Group A* *(Open hemorrhoidectomy)* *n = 110*	*Group B* *(Closed hemorrhoidectomy)* *n=103*	*P-value*
Operating time (min)	25.2+ 5.6 (15 – 35)	31.3 + 4.8 (25 – 40)	0.06†
Duration of hospitalization (days)	4.1+1.0 (3 – 5)	2.5+0.8 (2 – 4)	0.04†
Duration off work (days)	13.7+3.3 (10 – 21)	10.4+5.4 (7 – 18)	0.05†
Healing (days)	24.5 + 4.12 (20 – 25)	17 + 3.5 (10 – 20)	0.01†

† P value is statistically significant

*Results are expressed as Mean + Standard Deviation (Range)

Patients were evaluated for severity of pain when the effect of anesthesia was over. In both the procedures, no patient was pain free. Post operative pain scores were significantly low in the Group A than Group B during first 24 hours and at first bowel movements. (Table-III).

**Table-III:** Postoperative morbidity with open and closed techniques (n = 213).

	*Group A* *(Open hemorrhoidectomy)* *n = 110*	*Group B* *(Closed hemorrhoidectomy)* *n=103*	*P-value*
Postoperative pain score During the first 24 hours	6.12 + 1.5 (2-8)	5.0 + 0.02 (1-7)	< 0.0001^††^
Postoperative pain score At first bowel movements	4.5 + 1.5 (3-8)	5.0 + 1.6 (1-6)	0.02^†^
Retention of urine	13 (11.81%)	4 (3.88%)	0.01^†^
Reactionary haemorrhage	4 (3.63%)	-	NS
Wound healing 2 weeks	5 (4.54%)	27 (26.21%)	0.04^†^
Wound healing 3 weeks	31(28.18%)	77 (74.75%)	0.03^†^
Would dehiscence	-	3(2.91%)	NS
Recurrence	2(1.8%)	1 (0.97%)	NS
Anal stenosis	0	3 (2.91%)	NS
Anal incontinence	1(0.9%)	-	NS
Would infection	1(0.9%)	2(1.9%)	NS

NS = Not significant

^†^ P value is statistically significant

^††^ P value is statistically highly significant

*Results are expressed as Mean + Standard Deviation (Range)

** Results are presented as n(%)

 Mobility of patients was also assessed after both the procedures. In group A, out of 110 patients, 59 (53.63%) were mobile on 1^st^ postoperative day and 51 (46.36%) patients were mobile on 2^nd^ postoperative day and onwards. While in group B, out of 103 patients, 68 (66.01%) patients were mobile on the first postoperative day and 35 (33.98%) patients were mobile on 2^nd^ postoperative day and after wards, which is statistically significant p=0.05. 

 ost-operative complications were compared in both the techniques. Primary hemorrhage occurred in 4 (3.63%) patients of group A, no patient in group B developed this complication. Four patients (3.88%) in group B came with secondary hemorrhage, while in group A, no patient was seen having secondary hemorrhage. Retention of urine was seen in 13 (11.81%) patients in group A and 4 (3.88%) in group B. It was resolved with urinary catheterization in both groups. In both groups, no patient complained of faecal incontinence during six months follow-up. Four (3.63%) patients in group A came with recurrent hemorrhoids and in group B, only one (0.97%) patients reported recurrence. No patient in group A developed anal stenosis, while 3 (2.91%) patients in group B developed anal stenosis. They were managed conservatively by anal dilators for few weeks. Wound healing was significantly early in group B than group A. Wound infection was one (0.9%) in group A and two (1.9%) in group B.(Table-III).

## Discussion

 Hemorrhoids are universal and have been documented since ancient times. But their true incidence and etiology remains indecisive. Most patients with hemorrhoids remain asymptomatic. They only seek advice once they develop symptoms. The symptoms vary from bleeding to prolapse with or without discharge and itching. Definitive treatment is required for symptomatic hemorrhoids only.

 Many treatment modalities are available for haemorrhoids. These include rubber band ligation, cryosurgery, infrared coagulation and haemorrhoidectomy.^9^ Out of these modalities, most are performed in outpatient department and some are carried out as inpatient procedures under anesthesia.

 Hemorrhoids are very common in Pakistan and mostly the patients are reluctant to report to doctors and avoid to be examined by doctors for their anal and perianal conditions especially for females. So every bleeding per rectum is considered as due to hemorrhoids until proved otherwise. Treatment for hemorrhoids is started with laxatives, lubricants and blood stopping drugs even without examining the anal region. When the patients do not get relief and due to fear of dying they report to surgeons as a last resort. That is why we get the advanced cases of hemorrhoids and the rectal malignancy.

 Open excisional hemorrhoidectomy is the gold standard for third and fourth degree hemorrhoids. Milligan Morgan hemorrhoidectomy is easier to perform and is the most common operation performed in Pakistan for hemorrhoids. Ferguson hemorrhoidectomy is little bit difficult for the juniors to learn and perform and takes more time. Stapler hemorrhoidectomy may replace the open hemorrhoidectomy in future but due to high cost of Stapler gun it is not going to replace open hemorrhoidectomy in Pakistan.

 In this study, the results of open hemorrhoidectomy are compared with closed hemorrhoidectomy in patients with late 2^nd^ degree hemorrhoids not responding to non-surgical methods and 3^rd^ and 4^th^ degree hemorrhoids. The age of patients ranged from 22 to 70 years. The mean age was 47.5 years. Majority of patients i.e. 44.13% were aged between 41-50 years. This is also supported by other studies.^10^^,^^11^ Patients suffering from hemorrhoids complain of bleeding per rectum, prolapse, constipation, discharge and itching. In our study most patients presented with bleeding per rectum and prolapse. This is exactly same as described by other authors.^12^^,^^13^ Constipation although is not a symptom of hemorrhoids, but is an associated factor, since hemorrhoidal symptoms are aggravated by defecation, this results in hesitance of patients to pass stool and causes constipation. Out of 213 patients, 144 (67.60%) patients also complained of itching, this is due to soiling of perianal skin from discharge.

 Gencosmanoglu et al^2^ reported significantly shorter operating time with MMH than FH and this is also reflected in our study. Postoperative pain is a definitive outcome after hemorrhoidectomy due to the fact that anal canal lining is most abundantly innervated tissue in the digestive canal. The bare area of the anal canal following hemorrhoidectomy has been implicated as the cause of the pain. It is also related to anal sphincter spasm. Uncontrolled pain leads to prolonged hospital stay and delayed return to normal daily activities.^14^

 In this trial no significance different was found in term of pain complaints after hemorrhoidectomy in both groups. Similar results were also obtained by Carapeti et al^15^ in a randomized trial of open versus closed day case hemorrhoidectomy. Similarly, McConnell et al^16^ and Khubchandi^17^ reported same results. In another randomized trial Arbman et al.^18^ states that “although wound healing was considerably faster in patients operated on by the Ferguson technique there was no reduction in postoperative pain”. Whereas in this series, patients of group B who were operated by Ferguson technique complain of less pain than group A which were operated by Milligan-Morgan technique.

 Postoperative urinary retention as reported in literature is 2-36%^19^^,^^20^ and risk factors are spinal anesthesia, narcotic analgesics, male gender and intraoperative fluids used.^2^^,^^21^ In our series postoperative urinary retention was 3.88% in close group and 11.81% in open group.

 Postoperative bleeding reported in literature is in 0.6-5.4%^2^^,^^22^ patients while in our study there was no case in FH and 4 cases in MMH. The reoperation rate for bleeding is 0.4% in 30 days in literature^23^ but in our series two cases in MMH had to be operated again. Secondary hemorrhage occurred in 0.9% case in closed haemorrhoidectomy.

 Wound healing is another significant feature of the outcome of hemorrhoidectomy. Wound healing is secondary after MMH and large area of wound cause delayed healing and increased pain.^24^ The retraction of scars can lead to anal stenosis. The present study shows that MMH leads to longer healing time than FH .In our series three patients (2.9%) with anal stenosis required anal dilatation, while 2.55% is reported in literature. Arabman^18^ suggests fast wound healing in FH while Ho et al^25^ claim fast wound healing in MMH.

 Diathermy dissection has been reported as a probable cause of wound dehiscence, it may also increase the risk of infection.^26^ Ibrahim et al^27^ compared diathermy dissection with scissor excision for closed hemorrhoidectomy and found diathermy dissection leads to lower analgesic requirement than scissor excision. In this study patients had wound breakdown but exclusive of any major signs of infection or abscess; out of these three patients, two patients healed within three weeks and third in four weeks.

 Postoperative infection is claimed to be less after closed hemorrhoidectomy but the other school of thought says that the closure of wound increase the probability of septic complications.^28^ We found one in each group while McConnel and Khubchndani found none.^16^

 Hospital stay and the incapacity to work is more with MMH than FH in our series. Hosh et al^24^ also supports the same results while Gencosmanoglu^2^ et al shows the opposite results. In literature the incapacity to work was 20.2 days for MMH and 12.2 days for closed hemorrhoidectomy.^24^

 Recurrence of haemorrhoids was almost similar in both groups and is also supported in literature.^4^^,^^18^ Anal incontinence was not found in any group. We have low incidence of anal incontinence / leakage because we do not apply anal dilatation / sphincterotomy with both groups of hemorrhoidectomies.

## Conclusions

 Ferguson procedure or closed hemorrhoidectomy offered an advantage regarding postoperative pain, bleeding control, early mobility and rapid wound healing.
